# Smartphone Application for Celiac Patients: Assessing Its Effect on Gastrointestinal Symptoms in a Randomized Controlled Clinical Trial

**DOI:** 10.1155/2022/8027532

**Published:** 2022-07-08

**Authors:** Zeinab Nikniaz, Zahra Akbari Namvar, Masood Shirmohammadi, Elham Maserat

**Affiliations:** ^1^Liver and Gastrointestinal Diseases Research Center, Tabriz University of Medical Sciences, Tabriz, Iran; ^2^Student Research Committee, Tabriz University of Medical Sciences, Tabriz, Iran; ^3^Department of Health Information Technology, School of Health Management and Medical Informatics, Tabriz University of Medical Sciences, Tabriz, Iran

## Abstract

**Introduction:**

Considering the lack of inclusive Persian application for celiac patients that covers all aspects of the GFD, we developed a Persian-language application for patients with CD and assessed the effectiveness of a three-month educational intervention delivered via smartphone application compared with standard care on gastrointestinal symptom rating scale (GSRS) score in patients with celiac disease.

**Methods:**

In the present parallel randomized controlled clinical trial, 60 patients with CD were assigned randomly to receive education through a smartphone application (*n* = 30) or conventional clinical education (*n* = 30). The patients were asked to use it for getting the required information for three months. We assessed the gastrointestinal symptoms using the gastrointestinal symptom rating scale (GSRS) questionnaire at baseline and three months after interventions. The GSRS total score, celiac disease GSRS (CD-GSRS) score, abdominal pain, reflux, diarrhea, constipation, and indigestion scores were calculated.

**Results:**

Out of 60 randomized patients, 58 patients completed the study. In comparison to baseline, the mean score of CD-GSRS score (*p* = 0.001), and indigestion subscore (*p* < 0.001) were significantly decreased in the intervention group. The results of the between-group comparisons showed that there was a significant difference between the two groups only in the mean score of indigestion (*p* = 0.002).

**Conclusion:**

According to the results, using a smartphone application for providing information to patients with celiac disease had a significant positive effect on indigestion symptoms compared with routine clinic education. *Trial Registration*. This trial is registered with the Iranian registry of clinical trials (IRCT code: IRCT20170117032004N2; trial registry date: 2019.6.26).

## 1. Background

Celiac disease (CD) is an autoimmune entity characterized by gluten intolerance [1]. It is associated with the destruction of small intestine mucosa and is accompanied by different gastrointestinal (GI) and non-GI symptoms [1]. Eliminating gluten-containing foods from the diet is the only available treatment for these patients [2]. However, due to different reasons such as lack of knowledge, inadequate labeling, and high cost of gluten-free products, strict compliance to a gluten-free diet (GFD) is challenging [3]. So, studies indicated that providing dietary information to patients with CD could help them to increase their adherence to GFD [4–6]. Individualized education by an expert dietitian is considered the standard of care. However, it is costly and time-consuming. In this regard, other method of education such as group education was studied to overcome these limitations [4].

Recently, due to the global increase in the use of smartphones, transferring health messages through smartphones has grown. Mobile health (mHealth) is an appropriate approach to improving health outcomes using mobile technologies. This method is cost-effective, easy to use, and accessible [7, 8]. This method was used for promoting health-related behaviors in different studies [9–13]. In terms of diet therapy, the positive effects of a smartphone application in weight reduction programs [14–18] and nutrition education in different diseases such as diabetes [19], kidney diseases [20], and CVD [21] have been shown.

Considering the promising effect of smartphone applications in transferring nutritional information, we hypothesized that this method may also be an effective method of transferring GFD information to celiac patients.

Several applications were developed for patients with celiac disease. However, considering that in the field of dietetics, culturally appropriate nutrition guidelines and nutrition therapy recommendations are essential, and owing to the lack of inclusive Persian application for celiac patients that covers all aspects of the GFD, we developed a Persian-language application for patients with CD and confirmed its positive effect on patients' knowledge and adherence to the GFD [22]. In the present clinical trial, we aimed to study the effectiveness of a three-month educational intervention delivered via smartphone application compared with standard care on the gastrointestinal symptom rating scale (GSRS) score in patients with celiac disease. Moreover, in a post hoc analysis, the score of three domains that are most relevant to CD was compared between groups.

## 2. Literature Review

This literature review focuses on using smartphone applications for increasing the knowledge of patients with CD. The previous studies' structure is followed to compare the result of different studies on this topic [23, 24].

Mhealth technology was used in different studies to transfer health information to patients with different gastrointestinal diseases. A meta-analysis study has summarized the results of different studies on this topic and showed that GI eHealth interventions improved patients' QoL, psychological distress, medication adherence, and illness-related knowledge. In addition, these methods reduced the number of patient visits to the clinic/hospital. Of nineteen studies involved in the meta-analysis, only one was conducted on people with CD [5]. However, from then, different studies reported the effect of mhealth technology in patients with CD.

Sainsbury et al. in a clinical trial studied the effect of online education on knowledge and adherence to GFD in adult patients with CD. They showed that online education significantly increased GFD knowledge and adherence to GFD compared to the control group [5].

In another study on children with concurrent CD and type one diabetes, Connon et al. studied the effect of an interactive e-learning module on 18 children. The results showed that participants were “very satisfied” with this method. Knowledge test scores increased significantly from pre- to post-module completion [25]. Although the effectiveness of online education programs has been shown in different diseases, this method needs the Internet access to use. So, studies that used this method for transferring information had a high attrition rate (about 50%).

Haas et al. studied another method of mhealth technology in children with CD. In a randomized clinical trial, 61 children with CD received 45 text messages over three months, and the control group received standard of care treatment. Results indicated that the text message intervention significantly improved patients' quality of life. However, it has no significant effect on patients' adherence to the GFD [26]. The text message intervention had an important limitation, and very little amount of information can be transferred using this method.

Recently, application method is also developed for transferring information for patients with CD.

Plan My C-Day application has been developed to promote self-management skills among youth with CD [27].

Another mobile application has been developed to help patients with CD to share information regarding social, emotional, food, and wellness. This application had positive effect on patients' quality of life [28].

An English-language smartphone application, “MyHealthyGut,” was also developed for celiac patients [29], and its positive effect on gastrointestinal symptoms had been reported [30].

## 3. Method

### 3.1. Participants

In the present two-arm, parallel design randomized controlled clinical trial, the patients were selected randomly from the East-Azerbaijan celiac disease registry database, based on the following criteria: age>18 years, being at least six months post-diagnosis, and use a smartphone. The patients who had other chronic inflammatory GI diseases, diabetes, psychiatric, or neurological diseases that could interfere with assessments were excluded. All patients were diagnosed based on positive serology markers (including anti-tissue transglutaminase (tTG) antibodies, endomysial antibodies (EMA), and deamidated gliadin peptide (DGP) antibodies) confirmed by compatible duodenal histological findings.

At first “celiac application” is designed by our research team based on analysis, design, development, implementation, evaluation (ADDIE) model [31]. For this, we reviewed the published articles, various health, and diet-related applications and interviewed patients with celiac disease to gather the relative information to define the education scopes and purposes of the application and create the main contents and subcontents of the application. Then, based on this information, a professional web producer developed the first draft of an application. A team of expert gastroenterologists and nutritionists evaluated the first version. Besides, with the help of five patients with celiac disease, the usability of the application was checked. Based on the experts' and patients' comments, minor modifications were made, and the “celiac” was developed. This application is a free android-based application that can be used both on smartphones and android tab. It provides different information regarding CD and GFD in seven sections including “about the disease,” “gluten-free diet,” “gluten-free drugs and supplements,” “gluten-free food labeling,” “gluten-free cooking,” and “calculations” and “celiac centers in Iran.” In the “about the disease” section, various information such as disease causes, symptoms, diagnosis, and treatments were provided. In the “gluten-free diet” section, more than 150 foods were marked by three colors: green for allowed foods, red for prohibited foods, and orange for suspicious foods. The generic name and also factory name of the gluten-free drugs list which is introduced by Iran's food and drug organization were provided in the “gluten-free drugs and supplements” section. In the “gluten-free food labeling,” the names of the different food ingredients that may contain gluten were provided. In the “gluten-free cooking,” the cooking methods of different foods with gluten-free ingredients were provided. The calculation section allowed patients to calculate their body mass index (BMI) and also to register their tissue transglutaminase antibodies for follow-up. Finally, the “Celiac Disease Centers” section listed the address of CD clinics and gluten-free food providers in different provinces of Iran for more access for patients to gluten-free foods when they are on the journey. The patients were asked to use it for getting the required information for three months and informed us about the probable difficulties.

### 3.2. Patients Selection and Randomization

In the present study, 60 celiac patients were randomly selected out of 80 patients who had eligibility criteria. Patients were randomly assigned into two groups using the GraphPad randomization software tool. The randomization process was conducted by a third person not involved in the research protocol. All patients have received GFD recommendations in the celiac clinic of Imam Reza hospital with the help of leaflets before the study. An expert nutritionist designed the leaflets. The patients in the intervention group (*n* = 30) received the “celiac” application, and they were asked to use it for three months for getting the required information. The patients in the control group (*n* = 30) continued their weekly routine education with the help of leaflets in the clinic. Both programs (application or leaflet) had the identical topic and compromised information about the disease, gluten-free food items, gluten-free drugs, gluten-free food labeling, and also gluten-free cooking. The outcome assessor and statistician were blinded to group allocation.

### 3.3. Outcome Measure

The difference in the severity of gastrointestinal symptoms between the two groups was the outcome of interest that was assessed by the gastrointestinal symptom rating scale score (GSRS) questionnaire. This questionnaire is a seven-point Likert scale with 15 questions that has response options ranging from “no problem (0)” to “severe discomfort (6)”. The questionnaire is divided into 5 domains that cover the gastrointestinal system: diarrhea (3 questions; score range: 0-18), constipation (3 questions; score range: 0-18), abdominal pain (3 question; score range: 0-18), reflux (2 questions; score range: 0-12), and indigestion (4 questions; score range: 0-24). The questionnaire was translated into the Persian language and validated in our population previously [32]. In a post hoc analysis, the three domains that are most relevant to celiac disease were evaluated separately using celiac disease-GSRS (CD-GSRS). This modified questionnaire contains 10 questions from the GSRS and includes the following domains: diarrhea (3 questions; score range: 0-18), abdominal pain (3 questions; score range: 0-18), and indigestion (4 questions; score range: 0-24) [33]. Higher scores represent worse symptoms.

This study adheres to CONSORT guidelines.

### 3.4. Statistical Analysis

Originally, the study was designed and powered to detect the effects of a smartphone application on patients' knowledge and adherence level which were taken as the primary endpoints (data were presented in our previous publication). In this study, we presented the secondary outcome variables and the gastrointestinal effects of our intervention. Using the results of a previous study that assess the effect of education on celiac patients' adherence score (CDAT score) [26], assuming the significance level of 0.05 and power of 80%, the minimum sample size needed to detect a significant between-group difference in adherence level was 24 participants in each group. Considering the probability of a 20% attrition rate, the desired sample size was 30 patients in each group.

SPSS 21.0 was used for statistical analysis. Skewness and kurtosis were used for testing the assumption of normality. Considering the normal distribution of data, the continuous values were reported as mean and standard deviation, and the nominal and categorical variables such as sex, educational status, occupation categories, family history, comorbidities presence, and March categories were reported in frequency and percentage. Paired *t*-test was used to compare the changes in each GSRS score before and after the interventions. The differences in baseline values between the two groups were compared by the independent sample *t*-test. The one-way analysis of covariance (ANCOVA) with adjusting for age, sex, disease duration, educational level, and baseline values was used to compare the after intervention values between the two groups. For comparing the nominal and ordinal variables, the chi-square and Mann–Whitney *U* tests were used, respectively. Test results are reported as significant for *p* < 0.003, adjusted for multiplicity (Bonferroni's correction 0.05/14) [34].

## 4. Results

In the present study, out of 60 patients who were randomly divided into two groups, the questionnaire of one patient in the intervention group was incomplete, and one patient in the standard care group was lost to follow-up (Figure S1). The data were analyzed on 29 patients in each group.  Table 1 presents the baseline characteristics of patients. As indicated, the baseline characteristics were not significantly different between groups (*p* > 0.05).

**Figure 1:**
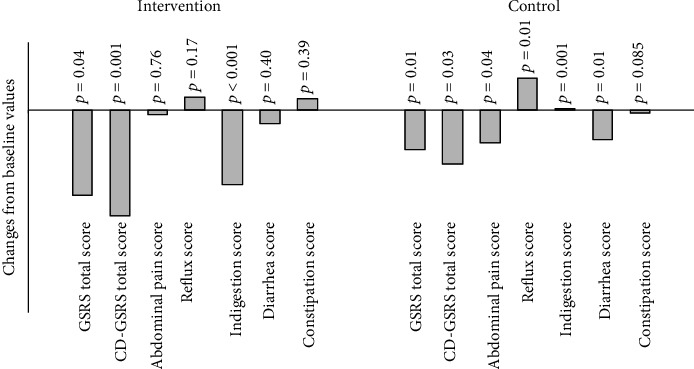
Mean changes from baseline of GSRS scores and subscores stratified by groups. CD: celiac disease. ∗*p* value of paired *t-*test comparing the mean changes from baseline of GSRS scores and subscores.

The mean changes from the baseline of GSRS scores and subscores stratified by groups are shown in Figure 1. As depicted, in comparison to baseline, the mean score of CD-GSRS score (*p* = 0.001) and indigestion subscore (*p* < 0.001) was significantly decreased in the intervention group. In the standard care group, compared to baseline, no significant changes were observed.

Table 2 shows the comparison of the mean GSRS total score and subscores between the two groups. There were no significant differences between the two groups at baseline. Three months after the intervention, the mean GSRS and CD-GSRS total scores were lower in the intervention group compared with the standard care group; however, the differences were not statistically significant. In the term of subscores, the indigestion score was significantly lower in the application group compared with the standard care group (*p* = 0.002).

None of the patients reported any problems regarding downloading or use of the application.

## 5. Discussion

Increasing the knowledge of celiac patients about gluten-free food, drugs, and commercial products was considered an important way of increasing their adherence to GFD and alleviating the disease symptoms [35]. In the present study, to overcome the limitations of the previous methods, we designed a smartphone application and assessed its efficacy in decreasing the GI symptoms in patients with celiac disease. We showed that a smartphone application was significantly more effective in decreasing indigestion score compared with the standard care group. These results were consistent with the findings of a previous study that used the “MyHealthyGut” smartphone application for celiac disease. Dowd et al. showed that one-month use of application had a significantly more positive effect on GI symptoms in celiac patients compared with the standard care group [30]. These findings may be due to the specific characteristics of smartphone applications that increase the patient's adherence to the diet. A large amount of information can be transferred through applications [36], and considering the mobility feature of them, by just installing the applications, they can be used whenever somebody needs them [37]. In addition to the gluten content of foods, other information, such as gluten-related labeling and gluten-free drug list, were also provided to patients through applications. Previously lack of knowledge about the gluten content of commercial foods and drugs was reported as a barrier to GFD adherence [35]. However, using this application, this information can easily be used during shopping and dining outside the home and could increase the patient's adherence to the diet.

Although GSRS total score and some subscores (reflux, abdominal pain, diarrhea, and constipation) were lower in the intervention group compared with the standard care group, they did not reach standard levels of significance. Although symptoms of celiac disease improve within weeks after strict adherence to GFD, not all patients have adequate adherence to GFD from the beginning of the study. So, we assume that a longer follow-up period would be associated with significant differences. Moreover, the possibility that the education via application was not superior to the standard care cannot be excluded.

## 6. Limitations

We acknowledge the limitations of the present study such as the short duration of the follow-up and low sample size. Moreover, assessing the GI symptoms was the secondary endpoint of this clinical trial, and the power calculation was not done for this aim. Also, we only included the patients who had the ability of reading and had a smartphone which limits its generalizability to all celiac patients.

## 7. Conclusions

In conclusion, the results showed that the “celiac” application was significantly more effective than routine clinic education in relieving indigestion symptoms. So, it can be used for increasing the GFD knowledge in patients with celiac disease. However, considering the limitations of the study, further investigations are needed to prove that providing information via a smartphones application would be sufficient to help patients feel better.

## 8. Future Work

Owing to the limitations of the present clinical trial, future trials should have a longer follow-up duration and larger sample sizes, including patients who had phones other than android phones, and assess gastrointestinal with more accurate methods.

## Figures and Tables

**Table 1:** The baseline characteristics of participants stratified by groups.

Variable	Intervention (*n* = 29)	Control (*n* = 29)	*p* value
Age (mean ± SD) years	36.04 ± 8.54	38.62 ± 9.88	0.28#
Sex n (%) male/female	8 (27.5)/21 (72.4)	10 (34.4)/19 (65.5)	0.22##
Education *n* (%)			
≤ Diploma	15 (51.72)	20 (68.9)	0.18##
College education	14 (48.27)	9 (31.0)	
Marital status			0.32##
Single	8 (27.58)	7 (24.1)	
Married	21 (72.4)	22 (75.8)	
Occupation			0.62##
Employed	10 (34.4)	11 (37.9)	
Student	3 (10.3)	3 (10.3)	
Housewife	16 (55.1)	15 (51.7)	
Positive family history	2 (6.8)	1 (3.4)	0.38##
Marsh			0.35##
I	4 (13.79)	3 (10.34)	
II	6 (20.68)	5 (17.24)	
IIIa	6 (20.68)	9 (31.03)	
IIIb	8 (27.58)	7 (24.13)	
IIIc	5 (17.24)	5 (17.24)	
Presence of comorbidities	6 (20.68)	9 (31.0)	0.55##
Disease duration (years)	5.32 ± 4.05	3.51 ± 2.26	0.16#

BMI: body mass index; SD: standard deviation. #*p* value of independent *t*-test. ##*p* value of chi-square.

**Table 2:** The comparison of the GSRS total scores and subscores between studied groups.

Variables	Application group (*n* = 29)		Placebo (*n* = 29)		*p* value#	*p* value##
	Before	After	Before	After		
GSRS total score	33.64 ± 18.44	27.28 ± 15.69	36.93 ± 23.47	35.09 ± 19.05	0.57	0.36
CD-GSRS	25.32 ± 13.62	17.48 ± 11.13	25.31 ± 16.92	22.09 ± 13.21	0.99	0.14
Abdominal pain	6.04 ± 4.76	5.04 ± 5.64	7.80 ± 6.01	5.65 ± 4.35	0.24	0.31
Reflux	3.04 ± 3.22	3.75 ± 4.02	3.93 ± 4.05	6.06 ± 4.22	0.38	0.09
Indigestion	13.84 ± 5.96	8.20 ± 5.3	13.23 ± 7.89	12.36 ± 7.91	0.40	0.002
Diarrhea	5.91 ± 7.16	4.62 ± 6.10	6.96 ± 6.75	4.89 ± 6.24	0.58	0.40
Constipation	5.62 ± 4.79	6.45 ± 6.97	8.46 ± 6.30	7.82 ± 7.92	0.07	0.47

GSRS: gastrointestinal symptom rating scale; CD-GSRS: celiac disease-gastrointestinal symptom rating scale. #*p* value of independent *t*-test. ##*p* value of one-way ANCOVA with adjusting to age, sex, disease duration, and baseline values.

## Data Availability

The datasets supporting the conclusions of this research will be available by request from the corresponding author, Zeinab Nikniaz.

## Abbreviations

CD: Celiac disease
GFD: Gluten-free diet
GSRS: Gastrointestinal symptom rating scale
CD-GSRS: Celiac disease-gastrointestinal symptom rating scale (GSRS)
ANCOVA: One-way analysis of covariance.

## Supplementary Materials

Supplementary Materials Figure S1 Flowchart for patient enrolment, randomization, and retention
