# Supplementary material for: Smartphone Application for Celiac Patients: Assessing Its Effect on Gastrointestinal Symptoms in a Randomized Controlled Clinical Trial
Source: Int J Telemed Appl. 2022 Jul 8;2022:8027532. doi: 10.1155/2022/8027532 (PMC9286948; doi:10.1155/2022/8027532)
Supplement: Supplementary Materials — Figure S1 Flowchart for patient enrolment, randomization, and retention [file 8027532.f1.docx]

Did not meet inclusion criteria

n=55

Assessed for eligibility (n=135)

Eligible patients (n=80)

Did not accept to participate

N=20

60 Randomized

Allocated to control group (n=30)

Allocation

Allocated to smartphone group (n=30)

Follow-up

Excluded (n=1)

Reason: Incomplete questionnaire

Excluded (n=1)

Reason: lost to follow-up

Analysis

Analysis (n=29)

Analysis (n=29)

**Figure S1:** Flow chart for patient enrolment, randomization, and retention
